# Sero-prevalence of Hepatitis B Virus Infection in Balochistan Province of Pakistan

**DOI:** 10.4103/1319-3767.80380

**Published:** 2011

**Authors:** Nadeem S. Sheikh, Azeem S. Sheikh, Aqleem A. Sheikh, Samira Yahya, Rafi-U-Shan, Majid Lateef

**Affiliations:** Department of Haematology & Transfusion Medicine, Bolan Medical College Complex, Quetta, Balochistan, Pakistan; 1 Department of Cardiology, Addenbrooke’s Hospital NHS Trust, Cambridge, UK; 2 Department of Gastroenterology, Shaikh Zayed Hospital, Lahore, Pakistan; 3 Department of Obstetrics & Gynaecology, Royal Free Hospital NHS Trust, London, UK; 4 AIDS Control Program, Balochistan, Pakistan; 5 Institute of Public Health, Quetta, Balochistan, Pakistan

**Keywords:** Cirrhosis, HBsAg, hepatitis B, hepatitis, prevalence

## Abstract

**Background/Aim:**

The objective was to evaluate the sero-prevalence of hepatitis B surface antigen (HBsAg) and IgM antibodies to hepatitis core antigen in Balochistan Province of Pakistan. Design of the study: A cross-sectional, population-based study. Place and time of the study: The study was conducted in Balochistan from 1^st^ January 2004 to 31^st^ December, 2008. The screening areas included Barkhan, Eashani, Khuzdar, Kodi Zikriani, Kohlu, Rakhni and Turbat.

**Materials and Methods:**

A total of 15,260 subjects were enrolled; 11,900 (78%) agreed to undergo screening. Fresh serum samples were tested for the presence of hepatitis B surface antigen and IgM antibodies to hepatitis B core antigen.

**Results:**

HBsAg was detected in 1166 (9.8%) while anti-HBc IgM was found in 117 (10.0%). HBsAg positivity was seen in 875 (12.7%) males and 291 (5.8%) females. The prevalence of hepatitis B in Balochistan varies from 3.3% in Khuzdar to 17.0% in Kodi Zikriani.

**Conclusions:**

It is utmost important to educate the public, to take proper measures to control the spread of infection and vaccination in order to interrupt transmission of this threatening public health problem in Balochistan province of Pakistan.

Hepatitis B Virus (HBV) occurs worldwide[1,2] and is an important public health problem which impacts the well being of more than 350 million people worldwide.[3,4]

The highest hepatitis B surface antigen (HBsAG) carrier rates are found in developing countries with primitive or limited medical facilities.[3] In areas of Africa and Asia, widespread infection may occur in infancy and childhood. The overall HBsAg carrier rates may be 10 to 15%.[1]

Adults infected with HBV usually acquire acute hepatitis B and recover, but 5 to 10% develop the chronic carrier state. Infected children rarely develop acute disease but 25 to 90% become chronic carriers. About 25% of carriers will die from cirrhosis or primary liver cancer as adults.[1,5] Cirrhosis and hepatocellular carcinoma account for more than 50% deaths in Asian men with chronic infection.[6]

The world can be divided into three areas on the basis of prevalence of chronic HBV infection: high (> 8%), intermediate (2 – 8%) and low (< 2%).[1,7]

High rates of hepatitis B virus infection in many South Asian countries are attributed to unsafe blood supply, re-use of contaminated syringes, lack of maternal screening to prevent perinatal transmission, and delay in introduction of hepatitis B vaccine.[8]

India, Pakistan, and Bangladesh have the highest rates of infection, with prevalence ranging from 2 to 8% in different population groups.[8]

The diagnosis of HBV infection is confirmed by demonstration in sera of specific antigens and/or antibodies. HBsAg can be detected in the serum from several weeks before onset of symptoms to months after onset. HBsAg is present in serum during acute infections and persists in chronic infections. The presence of HBsAg indicates that the person is potentially infectious.[1,9,10] The presence of HBeAg is associated with relatively high infectivity and severity of disease.[2,8]

Demonstration of IgM anti-HBc in serum indicates HBV infection, current or past. IgM anti-HBc is present in high titre during acute infection and usually disappears within six months although it can persist in some cases of chronic hepatitis. This test may, therefore, reliably diagnose acute HBV infection. IgG anti-HBc generally remains detectable for a lifetime.[1,2,9]

Acute hepatitis patients who maintain a constant serum HBsAg concentration or whose serum HBeAg persists eight to ten weeks after symptoms have resolved, are likely to become carriers and are at risk of developing chronic liver disease.[9]

This study was conducted as a population screening for chronic hepatitis B in the interior of Balochistan to know the brunt of the disease in these areas.

## MATERIALS AND METHODS

The study has been conducted as a cross-sectional, population-based screening in the interior of Balochistan between 1^st^ January 2004 and 31^st^ December, 2008. Keeping in view that the community data on the prevalence of HBsAg that are critical to define immunization strategies against hepatitis B virus infection are scarce, the present study has been designed. The sample size was provided by the Provincial office of the Federal Bureau of Statistics, who has the sampling frame of whole of Balochistan. Based on that sampling frame, they drew a multi-stage sample comprising 2270 households. The screening areas included the rural areas of Barkhan, Eeshani, Khuzdar, Kodi Zikriani, Kohlu, Rakhni, and Turbat. From these households, a total of 15,260 subjects were accessed for enrollment in this study, out of which 11,900 (78%) agreed to undergo screening. There was no difference of age, sex and area of 3360 (22%) patients who declined screening from those who agreed. The lowest response rates were seen in the older age groups (60 years or older- 8.7%). A total of 6874 (57.7%) males and 5026 (42.2%) females were screened. Median age was 34.5 years; SEM 1.79. A formal consent was taken from all the volunteers before sampling. The blood samples were collected by venipuncture using sterile disposable syringe. Appropriate controls were included to maintain quality control. The results were ethically revealed to the tested subjects. All positive cases were referred to the medical specialist for further management.

Fresh serum samples were tested for:

1. The presence of hepatitis B surface antigen on immunochromatography technique-based kit commercially available (Determine - Abbott Usa). All positive results were rechecked on HBsAg ELISA (Lab. System - Finland). False-positive results were seen in 154 (1.2%).
2. The presence of antibodies to hepatitis B core antigen.

The procedure was strictly followed as given in the kit. Negative and positive controls were run with each batch of samples. HBsAg positive subjects were further evaluated for IgM anti-HBc by ELISA (Human Germany).

Carrier of hepatitis B virus infection was defined as the presence of hepatitis B surface antigen and the absence of antibody to hepatitis B core antigen (IgM).

Screening for HBeAg as a measure of infectivity status was not conducted in this study.

The data was analyzed using the Statistical Package for Social Sciences (SPSS) Version 17.0 (SPSS Inc., IL, USA). *P* <0.05 was considered as statistically significant.

## RESULTS

Out of the total of 11900 subjects screened, HBsAg was detected in 1166 (9.8%) These surface antigen positive subjects of viral hepatitis were further tested for anti-HBc IgM, which was found reactive in 117 (10.0%). This shows that out of the total studied subjects, 1049 (8.8%) were actually chronic HBV carriers and only 117 (10.0%) subjects were having acute HBV infection.

HBsAg positivity was seen to be highest in Kodi Zikriani (17.0%) and lowest in Khuzdar (3.3%) of all the studied areas, as shown in Table 1.

**Table 1:** Area-specific seropositivity for HBsAg and anti-HBc IgM (n=11900)

Area	No. of screened subjects (%)	HBsAg positive No. (%:95% CI)	Anti-HBc IgM positive* No. (%: 95% CI)
Barkhan	982 (8.2)	119 (12.1 : 9.4 – 14.8)	22 (18.5 : 13.7 – 23.3)
Eeshani	1045 (8.7)	78 (7.5 : 6.3 – 8.7)	11 (14.1 : 12.4 – 15.8)
Khuzdar	2760 (23.1)	91 (3.3 : 2.7 – 3.9)	00.00
Kodi Zikriani	1075 (9.0)	183 (17.0 : 14.8 – 19.2)	32 (17.5 : 11.9 – 23.1)
Kohlu	3530 (29.6)	535 (15.1 : 13.9 – 16.3)	38 (7.1 : 5.4 – 8.8)
Rakhni	898 (7.5)	82 (9.1 : 7.8 – 10.4)	00.00
Turbat	1610 (13.5)	78 (4.8 : 3.4 – 6.2)	14 (18.0 : 16.9 – 19.1)
Total	11900	1166 (9.8 : 7.5 – 12.1)	117 (10.0: 8.2 –11.8)

* Calculated as percentage of HBsAg positive cases

The gender distribution of HBV infected subjects is shown in Table 2. A total of 6874 (57.7%) males and 5026 (42.2%) females were screened for HBV infection. HBsAg positivity was seen in 875 (12.7%) males 291 and (5.8%) females; anti-HBc IgM was positive in 76 (8.6%) males and 41 (14.0%) females highlighting an acute infection. The gender – HBsAg difference was statistically significant (*P* ≤ 0.000).

**Table 2:** Distribution of seropositive subjects by gender (n=11900)

Gender	Total screened (%)	HBsAg positive (%)	Anti-HBc IgM positive*(%)
Males	6874 (57.7)	875 (12.7)	76 (8.6)
Females	5026 (42.2)	291 (5.8)	41 (14)

* Calculated as percentage of HbsAg positive gender-specific cases

The age distribution of HBV infected subjects is shown in Table 3. The major bulk of HBsAg positive subjects, about 30%, consisted of subjects between 20 and 49 years of age. Comparing means shows that age – gender (*P* = 0.000) and age – HBsAg (*P* = 0.000) difference was statistically significant.

**Table 3:** Age-specific seropositivity for HBsAg and anti-HBc IgM (n= 11900)

Age group (years)	No. of screened subjects (%)	HBsAg positive (%)		Anti-HBc IgM positive* (%)	
		Male	Female	Male	Female
01-09	1907 (16.0)	53 (2.8)	15 (0.8)	9 (17.0)	4 (0.3)
10-19	1255 (10.5)	93 (7.4)	28 (2.2)	8 (8.6)	5 (17.8)
20-29	1849 (15.5)	224 (12.1)	68 (3.7)	13 (5.8)	6 (8.8)
30-39	2255 (19.0)	277 (12.2)	60 (2.6)	15 (5.4)	8 (13.3)
40-49	2142 (18.0)	151 (7.0)	71 (3.3)	18 (12.0)	10 (14.0)
50-59	1449 (12.1)	77 (5.3)	49 (3.4)	13 (16.8)	8 (16.3)
≥ 60	1043 (8.7)	00.00	00.00	00.00	00.00
Total	11,900	875 (7.3)	291 (2.4)	76 (8.8)	41 (14.0)

* Calculakted as percentage of HbsAg positive gender specific cases in each age-specific category

The age-specific risk factors identified in HBV infected subjects are shown in Table 4.

**Table 4:** Age-specific risk factors identified in HBV infected subjects (*n*=1166)

Age group (years)	Unknown (%)	Inoculation/ needle use (%)	Heterosexual/ extramarital (%)	IV drug use (%)	Blood transfusion (%)	Dialysis (%)	Previous surgery (%)
01-09	26 (2.2)	17 (1.45)	00.00	00.00	00.00	6 (0.5)	19 (1.6)
10-19	29 (2.4)	21 (1.8)	23 (2.0)	5 (0.4)	8 (0.7)	5 (0.4)	15 (1.3)
20-29	68 (5.8)	15 (1.3)	45 (3.8)	42 (3.6)	39 (3.3)	9 (0.7)	41 (3.5)
30-39	62 (5.3)	12 (1.0)	55 (4.7)	34 (2.9)	42 (3.6)	17 (1.45)	82 (7.0)
40-49	41 (3.5)	8 (0.7)	34 (2.9)	27 (2.3)	25 (2.1)	12 (1.0)	40 (3.4)
50-59	28 (2.4)	9 (0.7)	21 (1.8)	00.00	16 (1.3)	10 (0.8)	39 (3.3)
60 and above	00.00	00.00	00.00	00.00	00.00	00.00	00.00
Total	254 (21.6)	82 (7.0)	178 (15.2)	108 (9.2)	130 (11.0)	59 (4.8)	236 (20.1)

## DISCUSSION

Chronic viral hepatitis is one of the major public health problems in Pakistan. HBV has been reported in Pakistan with a prevalence rate of 10% in some studies.[11] In 2001–2002, Pakistan received a grant from the Global alliance for vaccines and immunization (GAVI) that has enabled the introduction of hepatitis B vaccination in routine Expanded program on immunization (EPI).[12] Vaccination for HBV as part of EPI was launched in a nationwide vaccination campaign in 2004.

According to the statistics obtained in this study, HBV infection has been detected in 9.8% of the subjects in the areas, out of which 10.0% had the acute infection, being positive both for HBsAg and IgM anti-HBc while 8.8% were chronic carriers, being positive for HbsAg only. Majority (≈50%) of the chronic HBV infected subjects were in the age group of 30-50 years. The carrier prevalence rates among males and females were 7.3% and 2.4%, respectively.

The prevalence of hepatitis B in the interior of Balochistan varies from as low as 3.3% in Khuzdar to as high as 17.0% in Kodi Zikriani. The situation in Barkhan, Eeshani, Kohlu and Rakhni is alarming with prevalence of 12.1%, 7.5%, 15.1% and 9.1% respectively. In another study by Marri and Ahmed, the prevalence of hepatitis B in Quetta was reported to be 11.0%.[13]

The exact factors responsible for having majority of chronic HBV infections in the age group of 30 to 50 years is not well understood in this part of the world. Reuse of syringes, unscreened blood transfusions, tattoo marks with unsterilized needles, sexual transmission from husband to wife and vice versa, and illegitimate personal contacts are some of the means of spreading of infections, as explored in the interview with the subjects.

Kammerlander and Zimmermann have reported 80% of HBV subjects between 15 and 40 years while mean age of 38 years has been reported in a study conducted in Chile, Santiago.[14,15] Sexual transmission, injection drug use and working in health care settings have been reported as the modes of HBV transmission in this age group.[16,17]

Hepatitis B virus causes a lot of morbidity and mortality in Pakistan. Prevention is the only way to control the disease by avoiding risk factors and observing all the necessary precautions while handling the infected blood and body fluids. A mass screening of HBV may be done in endemic areas in Balochistan and all those who are HBsAg negative should be vaccinated. Hepatitis B vaccine is safe, effective and well tolerated.[18]

WHO aims at controlling HBV worldwide to decrease the incidence of HBV-related chronic liver disease, cirrhosis, and hepatocellular carcinoma by integrating hepatitis B vaccination into routine infant (and possibly adolescent) immunization programs.[1,5,10]

In 1991, the Global Advisory Group of EPI (Extended Programme on Immunization) set 1997 as the target for integrating the hepatitis B vaccination into national immunization programs worldwide. The group recommended strategies for implementation and delivery that vary according to epidemiology: advocating integration of the vaccine into immunization programs by 1995 in countries with HBV carrier prevalence of 8% or higher and setting 1997 as the target date for all other countries. WHO endorsed the recommendation in May 1992 and the World Health Assembly added a disease reduction target for hepatitis B in 1994, calling for an 80% decrease in new HBV child carriers by 2001.

Attaining global immunization coverage is a goal still unmet. Attempts at protecting the whole community by vaccinating only high-risk individuals have not been successful.[19] Universal vaccination is necessary to control and possibly eradicate hepatitis B.

Screening of blood prior to transfusion and vaccination of the infants for HBV as part of the nationwide EPI campaign would help in limiting the spread of this infectious disease which has been one of the major causes of morbidity and mortality in this part of the world.

Commitment of public health resources to eliminate the spread of HBV requires recognition of the importance of hepatitis B, persistent efforts to ensure that populations are protected, and patience to achieve goals of disease reduction.[1]
